# A randomized, open-label trial of edoxaban in Japanese patients with severe renal impairment undergoing lower-limb orthopedic surgery

**DOI:** 10.1186/s12959-014-0034-9

**Published:** 2015-01-30

**Authors:** Takeshi Fuji, Satoru Fujita, Yohko Kawai, Yasuyuki Abe, Tetsuya Kimura, Masayuki Fukuzawa, Kenji Abe, Shintaro Tachibana

**Affiliations:** Department of Orthopaedic Surgery, Japan Community Healthcare Organization, Osaka Hospital, 4-2-78 Fukushima, Fukushima-ku, Osaka 553-0003 Japan; Department of Orthopaedic Surgery, Takarazuka Daiichi Hospital, Takarazuka, Japan; International University of Health and Welfare, Tokyo, Japan; Department of Orthopaedic Surgery, Kumamoto Chuo Hospital, Kumamoto, Japan; Clinical Planning Department, Daiichi Sankyo Co. Ltd, 1-2-58, Hiromachi, Shinagawa-ku, Tokyo, 140-8710 Japan; Clinical Execution Department, Daiichi Sankyo Co. Ltd, 1-2-58, Hiromachi, Shinagawa-ku, Tokyo, 140-8710 Japan; Clinical Data & Biostatistics Department, Daiichi Sankyo Co. Ltd, 1-2-58, Hiromachi, Shinagawa-ku, Tokyo, 140-8710 Japan; Department of Orthopaedic Surgery, Mishuku Hospital, Tokyo, Japan

**Keywords:** Edoxaban, Renal impairment, Thromboprophylaxis, Orthopedic surgery

## Abstract

**Background:**

Edoxaban is an oral, direct, factor Xa inhibitor approved in Japan for thromboembolic prophylaxis after lower-limb orthopedic surgery (LLOS), but contraindicated in patients with severe renal impairment (SRI; creatinine clearance [CL_CR_] ≥15 to <30 mL/min).

**Methods:**

This open-label study compared the safety of edoxaban 15 mg once daily in Japanese patients with SRI to that of edoxaban 30 mg in patients with mild renal impairment (MiRI; CL_CR_ ≥50 to ≤80 mL/min; N = 30) undergoing LLOS. Patients with CL_CR_ ≥20 to <30 mL/min were randomized to receive edoxaban 15 mg (N = 22) or subcutaneous fondaparinux 1.5 mg once daily (N = 21). All patients with CL_CR_ ≥15 to <20 mL/min received edoxaban 15 mg (N = 7). Treatment was administered for 11 to 14 days.

**Results:**

Major or clinically relevant non-major bleeding occurred in 6.7%, 3.4%, and 5.0% of patients in the MiRI edoxaban 30-mg, SRI edoxaban 15-mg, and SRI fondaparinux groups, respectively; there were no major bleeding events. No thromboembolic events occurred. At all time points assessed, edoxaban plasma concentrations and changes in coagulation biomarkers were similar between the SRI and MiRI groups.

**Conclusions:**

These results suggest edoxaban 15 mg once daily is well tolerated in Japanese patients with SRI undergoing LLOS.

**Trial registration:**

Clinicaltrials.gov Identifier: NCT01857583.

**Electronic supplementary material:**

The online version of this article (doi:10.1186/s12959-014-0034-9) contains supplementary material, which is available to authorized users.

## Background

Lower-limb orthopedic surgery (LLOS), including total knee arthroplasty (TKA), total hip arthroplasty (THA), and hip fracture surgery (HFS), is considered a high risk factor for the development of venous thromboembolism (VTE). As such, current guidelines for the prevention of VTE, including those from the American College of Chest Physicians and the Japanese Circulation Society, recommend anticoagulation therapy for VTE prevention in patients undergoing LLOS [1-4]. Anticoagulants currently approved in Japan for VTE prevention in patients undergoing LLOS include the subcutaneous low-molecular weight heparin (LMWH) enoxaparin; subcutaneous fondaparinux; orally administered warfarin; and the oral, direct factor Xa inhibitor edoxaban [5-8].

In Japan, the majority of patients undergoing LLOS are ≥70 years of age [9]; older individuals are at increased risk for developing renal impairment [10]. Currently, there are limited therapeutic options available in Japan for the prevention of VTE following LLOS in patients with severe renal impairment (SRI; creatinine clearance [CL_CR_] ≥15 to <30 mL/min). Enoxaparin is contraindicated in patients with CL_CR_ <30 mL/min [5] and fondaparinux is not approved for use in patients with CL_CR_ <20 mL/min [6]. Warfarin also is contraindicated in patients with serious renal impairment [8].

Edoxaban is a novel, oral, highly specific and direct inhibitor of factor Xa, which is a key serine endopeptidase of the coagulation cascade [11]. Edoxaban has predictable pharmacokinetics (PK), a rapid onset of action with approximately 62% oral bioavailability [12], and low intrasubject variability [13]. Approximately 50% of the absorbed edoxaban dose is renally eliminated [12]. Currently, edoxaban 30 mg once daily is approved in Japan for the prevention of VTE in patients undergoing LLOS but is contraindicated in patients with SRI [7]. A population pharmacometric analysis of pooled data from edoxaban phase 1 and 2 clinical trials, including a phase 2b study in patients undergoing THA [14], revealed that CL_CR_ was a significant predictor of edoxaban clearance (decreasing renal function decreases renal clearance), and surgery significantly slowed the rate of edoxaban absorption [15]. This analysis revealed a direct, linear relationship between edoxaban exposure and efficacy but a relationship to bleeding was not identified [15].

The objective of this study was to evaluate the safety of orally administered edoxaban at a reduced dose of 15 mg in Japanese patients with SRI (CL_CR_ ≥15 to <30 mL/min) compared with edoxaban 30 mg in patients with mild renal impairment (MiRI; CL_CR_ ≥50 to ≤80 mL/min) and to subcutaneous fondaparinux 1.5 mg in patients with SRI (CL_CR_ ≥20 to <30 mL/min) undergoing LLOS. In addition, edoxaban plasma concentrations, biomarkers of coagulation, and efficacy were assessed.

## Methods

### Study design

**Figure 1:**
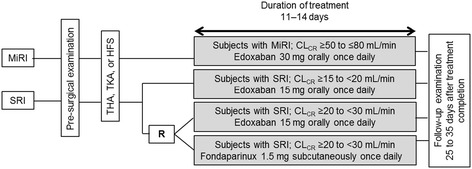
**Study design.** CL_CR_ = creatinine clearance; HFS = hip fracture surgery; MiRI = mild renal impairment; R = randomization; SRI = severe renal impairment; THA = total hip arthroplasty; TKA = total knee arthroplasty.

This was a multicenter, open-label, 3-parallel-group, phase 3 study in Japanese patients undergoing LLOS (NCT01857583). Patients with MiRI (CL_CR_ ≥50 to ≤80 mL/min) received oral edoxaban 30 mg once daily. Among those with SRI, patients with a CL_CR_ ≥20 to <30 mL/min, were randomized to either oral edoxaban 15 mg once daily or subcutaneous fondaparinux 1.5 mg once daily; patients with a CL_CR_ ≥15 to <20 mL/min received edoxaban 15 mg once daily. Fondaparinux was included as a comparator because it is approved in Japan for thromboprophylaxis in patients undergoing LLOS with SRI at a CL_CR_ ≥20 to <30 mL/min. Edoxaban was administered orally 12 to 24 hours after surgery and fondaparinux subcutaneously ≥24 hours after surgery. Both edoxaban and fondaparinux were administered for 11 to 14 days. Follow-up examination was performed at 25 to 35 days after the last dose of study drug (Figure 1). Concomitant physiotherapy (intermittent pneumatic compression devices or elastic stockings) was permitted throughout the treatment period.

Patients discontinued treatment if they developed symptomatic deep vein thrombosis (DVT), pulmonary embolism (PE), or major bleeding; had evidence of hepatic dysfunction; had persistent systolic blood pressure of >180 mm Hg or diastolic blood pressure of >110 mm Hg; required anticoagulant drugs, antiplatelet drugs, thrombolytic drugs, or drugs that might affect thrombus formation; needed postsurgical epidural anesthesia; initiated hemodialysis; experienced an adverse event (AE) that was judged sufficient by the investigator for study discontinuation; voluntarily requested to withdraw; were found to be ineligible for continued participation in the study for any reason; or if the investigator decided that study participation should be discontinued for any other reason.

This study was conducted in compliance with the Declaration of Helsinki, Good Clinical Practice (GCP) regulations and guidelines, and all applicable local regulations. All study protocols, information for patients, and informed consent forms received approval from an institutional review board.

### Patient selection

Eligibility criteria included patients who were ≥20 years of age, had SRI or MiRI, and were undergoing unilateral TKA or THA (excluding revision surgeries), or HFS for medial or lateral femoral neck fracture (trochanteric or subtrochanteric section of the femur) within 10 days of the presurgical examination. Informed consent was obtained from all patients. Presurgical exclusion criteria included, but were not limited to, patients undergoing or possibly undergoing hemodialysis; risk of bleeding; risk of thromboembolism; and hepatic dysfunction. Postsurgical exclusion criteria included, but were not limited to, CL_CR_ <15 mL/min; abnormal bleeding at the site of spinal anesthesia; abnormal or excessive bleeding during or immediately after surgery; and the inability to take oral medication.

### Endpoints

At the presurgical evaluation, pretreatment (postsurgery), on day 7, on the completion day of treatment, and during the follow-up examination, the following procedures were performed: blood pressure, pulse rate, blood sample collection for hematology and blood biochemistry tests, and urinalysis. The occurrence of thromboembolic events, bleeding, and all other adverse events (AEs) were recorded throughout the study.

Safety endpoints included the incidences of any bleeding event (major, clinically relevant non-major [CRNM], or minor bleeding); major or CRNM bleeding; major bleeding; CRNM bleeding; minor bleeding; AEs; and adverse drug reactions (ADRs). Major bleeding was defined as fatal bleeding; clinically overt bleeding accompanied by a decrease in hemoglobin of >2 g/dL or requiring a transfusion of >4 units of blood (1 unit = ~200 mL); retroperitoneal, intracranial, intraocular, or intrathecal bleeding; or bleeding requiring repeat surgery. Clinically relevant non-major bleeding was defined as bleeding that could not be categorized as major bleeding but corresponded to a hematoma of ≥5 cm in diameter; epistaxis or gingival bleeding that occurred in the absence of external factors and lasted ≥5 min; gastrointestinal bleeding; gross hematuria that persisted for >24 h; or other bleeding determined by the investigator to be clinically significant. Minor bleeding was defined as all other bleeding events that did not fall under the categories of major or CRNM bleeding. All bleeding events were reassessed by a bleeding event assessment committee in a blinded manner. Adverse events were summarized by system organ class and preferred term according to the Medical Dictionary for Regulatory Activities (Version 15.1).

The efficacy endpoint was the incidence of symptomatic VTE (composite of symptomatic DVT or PE) during the treatment period. Venography was not used in this study because a contrast agent cannot be used in patients with SRI; therefore, only symptomatic VTE was assessed. If symptomatic DVT was suspected, it was confirmed by ultrasonography or computed tomography (CT) scan. Suspected symptomatic PE was confirmed by ultrasonography, pulmonary scintigraphy, CT scan, or chest X-ray. All thromboembolic events were assessed by a thromboembolic event assessor, blinded to treatment group, based on imaging results.

### Plasma concentration and biomarker assessments

Blood samples for the determination of plasma edoxaban concentrations were obtained at predose, 1 to 3 hours postdose, 4 to 8 hours postdose on day 7, and on the completion day of study treatment or at the time of discontinuation. Edoxaban plasma concentrations were measured using a validated liquid chromatography separation method with tandem mass detection methods (Advion BioServices, Inc; Ithaca, NY).

Blood samples for the measurement of biomarkers of coagulation were obtained at the presurgical examination, at predose, 1 to 3 hours postdose, 4 to 8 hours postdose on day 7, and on the completion day of study treatment or at the time of discontinuation. Biomarkers assessed were prothrombin time (PT) and activated partial thromboplastin time (aPTT). Presurgical measures of PT and aPTT were performed at the study site (in-hospital) as part of the presurgical examination and determination of eligibility for enrollment. Biomarker measurements postsurgery and during the treatment period were performed at a central laboratory (SRL, Inc; Hachioji, Japan).

### Statistical analysis

All patients who were enrolled, received study drug, had safety data after the start of study treatment, and had no significant GCP violations were included in the safety analysis set. The incidence and corresponding 95% confidence intervals (CIs) of any bleeding, major or CRNM bleeding, and individual bleeding events (major, CRNM, and minor) that occurred from the start of study treatment to the day following the end of study treatment were calculated for each treatment group. For the analyses of any bleeding and major or CRNM bleeding, the absolute difference and corresponding 95% CIs were calculated between patients with SRI who received edoxaban 15 mg and those with MiRI who received edoxaban 30 mg, as well as between patients with CL_CR_ ≥20 to <30 mL/min who received edoxaban 15 mg and fondaparinux 1.5 mg. Additional safety endpoints (AEs, serious AEs, AEs leading to treatment discontinuation, and adverse drug reactions [ADRs]) were summarized according to incidence by treatment group and corresponding 95% CIs.

Data from patients in the safety analysis set with valid plasma drug concentration data measured at ≥1 time points were included in descriptive statistics. Descriptive statistics for edoxaban plasma drug concentrations were calculated by time point and the severity of renal impairment (SRI and MiRI). In patients with SRI, descriptive statistics were also calculated by time point and according to CL_CR_ level (CL_CR_ ≥15 to <20 mL/min, CL_CR_ ≥ 20 to <30 mL/min).

Data from patients in the safety analysis set with valid biomarker data measured at ≥1 time points were included in descriptive statistics. Descriptive statistics of measured biomarkers were calculated by treatment group at each time point. Scatter plots were generated to determine the relationship between plasma edoxaban concentrations versus PT and aPTT using individual data values from day 7 and the completion day of treatment.

The efficacy analysis set included all patients who were enrolled in the study, received study drug, and had no significant GCP violations. The incidence of VTE (composite of symptomatic DVT and symptomatic PE), symptomatic DVT, symptomatic PE, VTE-related deaths, and their respective 95% CIs were calculated by treatment group.

A total of 80 patients undergoing TKA, THA, or HFS were to be enrolled, comprising 30 patients with MiRI to receive edoxaban 30 mg, 30 patients with SRI to receive edoxaban 15 mg, and 20 patients with SRI to receive fondaparinux 1.5 mg. The sample size was based on integrated analyses from previous phase 2 and phase 3 studies of edoxaban in patients undergoing TKA, THA, or HFS [16-22]. These studies demonstrated that, in patients with MiRI who were administered edoxaban 30 mg once daily, the incidence of any bleeding and major or CRNM bleeding was 22.8% and 5.0%, respectively. The proposed sample size of 80 patients provides 95% or higher probability of detection of major or CRNM bleeding in ≥1 patient assuming that the incidence of major or CRNM bleeding in patients with SRI receiving edoxaban 15 mg is more than twice that in patients with MiRI.

## Results

### Patient disposition

**Figure 2:**
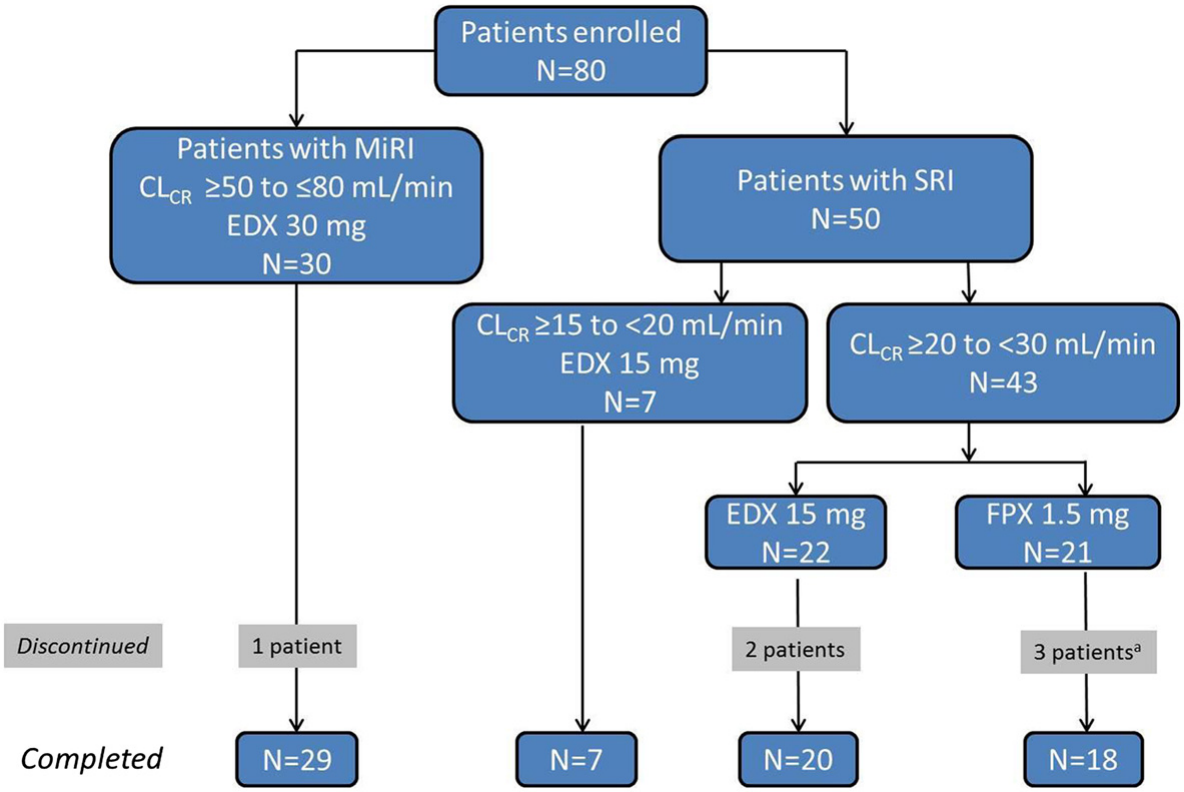
**Patient disposition.** CL_CR_ = creatinine clearance; EDX = edoxaban; FPX = fondaparinux; MiRI = mild renal impairment; SRI = severe renal impairment. ^a^One patient randomized to FPX 1.5 mg discontinued prior to initiation of study treatment due to excessive bleeding from the surgical wound.

A total of 80 patients were enrolled. Of the 50 patients with SRI, 43 had a CL_CR_ of ≥20 to <30 mL/min and received either 15 mg edoxaban (n = 22) or 1.5 mg fondaparinux (n = 21), while 7 patients with a CL_CR_ of ≥15 to <20 mL/min received edoxaban 15 mg. All 30 patients with MiRI received edoxaban 30 mg once daily (Figure 2). One patient randomized to the fondaparinux treatment group received edoxaban 15 mg in error, while a second patient in the fondaparinux group discontinued prior to initiation of study treatment. A total of 74 patients completed the study, as 6 had discontinued study treatment (1 patient with MiRI and 5 with SRI). Reasons cited for discontinuation included AE occurrence, voluntary withdrawal, and ineligibility for continued participation.

### Patient demographics and baseline characteristics

The mean age of patients ranged from 78 to 92 years across the treatment groups. The majority of patients were female (91%), ≥75 years of age (87%), and weighed ≤60 kg (86%). The majority of patients in each treatment group underwent HFS: 58% of patients overall. All patients received concomitant physiotherapy. Compared with patients with MiRI, patients with SRI were older (mean age 78 vs 88 years, respectively) and had a lower mean body weight (55 vs 43 kg, respectively). A mean body weight of ≤60 kg was present in 97% of the patients receiving edoxaban 15 mg in the SRI group. Concomitant use of nonsteroidal anti-inflammatory drugs was comparable among patients receiving edoxaban or fondaparinux and use of P-gp inhibitors was low (Table 1).

**Table 1:** **Patient demographics and baseline characteristics**

		**SRI**			**MiRI**
		**Edoxaban 15 mg**	**Edoxaban 15 mg**	**Fondaparinux 1.5 mg**	**Edoxaban 30 mg**
**CL**_**CR**_ **(mL/min)**		**≥15 to <20**	**≥20 to <30**	**≥20 to <30**	**≥50 to ≤80**
		**N = 7**	**N = 22**	**N = 20**	**N = 30**
Female, *n (%)*		6 (85.7)	19 (86.4)	20 (100.0)	27 (90.0)
Age, yr					
*mean (SD)*		91.7 (7.1)	86.5 (4.3)	85.7 (8.9)	78.1 (6.5)
<75, *n (%)*		0	0	2 (10.0)	8 (26.7)
≥75, *n (%)*		7 (100.0)	22 (100.0)	18 (90.0)	22 (73.3)
Weight, kg					
*mean (SD)*		39.4 (3.8)	44.3 (8.9)	46.1 (8.0)	54.5 (11.1)
≤60, *n (%)*		7 (100.0)	21 (95.5)	18 (90.0)	22 (73.3)
>60, *n (%)*		0	1 (4.5)	2 (10.0)	8 (26.7)
CL_CR_, mL/min					
*median*		18.7	27.6	26.2	62.7
*min–max*		15.3–20.0	20.4–30.0	22.8–30.0	50.7–79.8
Surgery, *n (%)*					
TKA		0	5 (22.7)	6 (30.0)	9 (30.0)
THA		0	1 (4.5)	4 (20.0)	8 (26.7)
HFS		7 (100.0)	16 (72.7)	10 (50.0)	13 (43.3)
Physiotherapy use, *n (%)*					
Present		7 (100.0)	22 (100.0)	20 (100.00)	30 (100.0)
Elastic stocking		7 (100.0)	21 (95.5)	19 (95.0)	23 (76.7)
Intermittent pneumatic compression		5 (71.4)	8 (36.4)	9 (45.0)	19 (63.3)
Concomitant use of NSAIDs, *n (%)*		6 (85.7)	20 (90.9)	18 (90.0)	29 (96.7)
Concomitant use of P-gp inhibitor, *n (%)*		1 (14.3)	0 (0.0)	0 (0.0)	1 (3.3)

CL_CR_ = creatinine clearance (calculated using the Cockcroft-Gault formula); HFS = hip fracture surgery; MiRI = mild renal impairment; NSAIDs = nonsteroidal anti-inflammatory drugs; P-gp = P-glycoprotein; SRI = severe renal impairment; THA = total hip arthroplasty; TKA = total knee arthroplasty.

### Bleeding events

The incidence of any bleeding event occurred in 10 (33.3%) patients with MiRI receiving edoxaban 30 mg compared with 6 (20.7%) patients with SRI receiving edoxaban 15 mg (absolute difference 12.6% [95% CI: −10.0, 33.6]) and 8 (40.0%) patients with SRI receiving fondaparinux. Among patients with CL_CR_ ≥20 to <30 mL/min, the incidence of any bleeding event trended higher for those receiving fondaparinux 1.5 mg (40.0%) compared with those receiving edoxaban 15 mg (22.7%), for an absolute difference of 17.3% (95% CI, −10.2, 42.1). Major or CRNM bleeding was reported in 2 (6.7%) patients with MiRI receiving edoxaban 30 mg, 1 (3.4%) patient with SRI receiving edoxaban 15 mg, and 1 (5.0%) patient with SRI receiving fondaparinux. All of these were CRNM bleeding events (Table 2). The difference in rates (95% CI) of major or CRNM bleeding between patients with MiRI and SRI receiving edoxaban was 3.2% (95% CI, −11.3, 18.1). Among patients with CL_CR_ ≥20 to <30 mL/min, the incidence of major or CRNM bleeding in those receiving fondaparinux 1.5 mg was 5.0% compared with 4.5% in patients in the edoxaban 15-mg group, resulting in an absolute difference of 0.5% (95% CI, −17.3, 19.4). The single CRNM bleeding event in the SRI edoxaban group was a subcutaneous hematoma in a patient with CL_CR_ ≥20 to <30 mL/min. A wound hematoma was reported as the single CRNM bleeding event in the fondaparinux treatment group. In the MiRI group, the 2 reported CRNM bleeding events were hematuria and subcutaneous hematoma. Minor bleeding occurred in 10 (33.3%) patients with MiRI receiving edoxaban 30 mg, 5 (17.2%) patients with SRI receiving edoxaban 15 mg, and 7 (35.0%) patients with SRI receiving fondaparinux (Table 2).

**Table 2:** **Incidence of bleeding events during the treatment period** ^**a**^

	**MiRI**	**SRI**			
	**Edoxaban 30 mg**	**Edoxaban 15 mg**			**Fondaparinux 1.5 mg**
**CL**_**CR**_ **mL/min**	≥50 to ≤80	≥15 to <30	≥15 to <20	≥20 to <30	≥20 to <30
***N***	30	29 (total)	7	22	20
**Any bleeding events**					
*n (%)*	10 (33.3)	6 (20.7)	1 (14.3)	5 (22.7)	8 (40.0)
*95% CI* ^*b*^	19.2, 51.2	9.8, 38.4	2.6, 51.3	10.1, 43.4	21.9, 61.3
**Major or CRNM bleeding**					
*n (%)*	2 (6.7)	1 (3.4)	0	1 (4.5)	1 (5.0)
*95% CI* ^*b*^	1.8, 21.3	0.6, 17.2	0.0, 35.4	0.8, 21.8	0.9, 23.6
**Major bleeding**					
*n (%)*	0	0	0	0	0
*95% CI* ^*b*^	0.0, 11.4	0.0, 11.7	0.0, 35.4	0.0, 14.9	0.0, 16.1
**CRNM bleeding**					
*n (%)*	2 (6.7)	1 (3.4)	0	1 (4.5)	1 (5.0)
*95% CI* ^*b*^	1.8, 21.3	0.6, 17.2	0.0, 35.4	0.8, 21.8	0.9, 23.6
**Minor bleeding**					
*n (%)*	10 (33.3)	5 (17.2)	1 (14.3)	4 (18.2)	7 (35.0)
*95% CI* ^*b*^	19.2, 51.2	7.6, 34.5	2.6, 51.3	7.3, 38.5	18.1, 56.7

CI = confidence interval; CRNM = clinically relevant non-major; MiRI = mild renal impairment; SRI = severe renal impairment.

^a^Safety analysis set; ^b^Score method.

### Adverse events

The incidence of AEs was 73.3% in patients with MiRI receiving edoxaban 30 mg compared with 62.1% in patients with SRI receiving edoxaban 15 mg (Table 3). Among patients with CL_CR_ ≥20 to <30 mL/min, AEs occurred in 12 patients in each group receiving edoxaban 15 mg (54.5%) or fondaparinux 1.5 mg (60.0%). Subcutaneous hematoma, blood in the urine, cystitis, and increases in γ-glutamyltransferase were reported more frequently in the edoxaban groups than in the fondaparinux group. In addition, increases in alanine aminotransferase (ALT), aspartate aminotransferase (AST), and blood alkaline phosphatase levels were all reported more frequently in the SRI edoxaban 15-mg group compared with the MiRI edoxaban 30-mg group or the SRI fondaparinux group. No patients experienced elevations in ALT or AST levels ≥3× the upper limit of normal (ULN) or in total bilirubin levels ≥2× ULN.

**Table 3:** **Adverse events**

	**MiRI**	**SRI**			
	**Edoxaban 30 mg**	**Edoxaban 15 mg**			**Fondaparinux 1.5 mg**
**CL**_**CR**_ **mL/min**	≥50 to ≤80	≥15 to <30	≥15 to <20	≥20 to <30	≥20 to <30
***N***	30	29 (total)	7	22	20
**Adverse events**					
*n (%)*	22 (73.3)	18 (62.1)	6 (85.7)	12 (54.5)	12 (60.0)
*95% CI* ^*a*^	55.6, 85.8	44.0, 77.3	48.7, 97.4	34.7, 73.1	38.7, 78.1
**MedDRA preferred term, reported by ≥2 patients,** ***n (%)***					
Alanine aminotransferase increased	2 (6.7)	3 (10.3)	1 (14.3)	2 (9.1)	0 (0.0)
Aspartate aminotransferase increased	1 (3.3)	5 (17.2)	2 (28.6)	3 (13.6)	0 (0.0)
Blood alkaline phosphatase increased	0 (0.0)	6 (20.7)	1 (14.3)	5 (22.7)	0 (0.0)
Blood bilirubin increased	2 (6.7)	0 (0.0)	0 (0.0)	0 (0.0)	0 (0.0)
Blood lactate dehydrogenase increased	0 (0.0)	2 (6.9)	1 (14.3)	1 (4.5)	0 (0.0)
Blood urine present	4 (13.3)	2 (6.9)	0 (0.0)	2 (9.1)	1 (5.0)
Cystitis	2 (6.7)	5 (17.2)	2 (28.6)	3 (13.6)	1 (5.0)
Gamma-glutamyltransferase increased	5 (16.7)	6 (20.7)	2 (28.6)	4 (18.2)	0 (0.0)
Hemorrhage subcutaneous	6 (20.0)	1 (3.4)	0 (0.0)	1 (4.5)	3 (15.0)
Hemoglobin decreased	0 (0.0)	1 (3.4)	1 (14.3)	0 (0.0)	3 (15.0)
Nasopharyngitis	0 (0.0)	2 (6.9)	0 (0.0)	2 (9.1)	1 (5.0)
Red blood cells urine positive	2 (6.7)	0 (0.0)	0 (0.0)	0 (0.0)	0 (0.0)
Subcutaneous hematoma	1 (3.3)	3 (10.3)	1 (14.3)	2 (9.1)	0 (0.0)
Urinary tract infection	2 (6.7)	1 (3.4)	0 (0.0)	1 (4.5)	2 (10.0)
**Adverse drug reactions**					
*n (%)*	13 (43.3)	7 (24.1)	1 (14.3)	6 (27.3)	6 (30.0)
*95% CI* ^*a*^	27.4, 60.8	12.2, 42.1	2.6, 51.3	13.2, 48.2	14.5, 51.9
**Serious adverse events**					
*n (%)*	1 (3.3)	5 (17.2)	3 (42.9)	2 (9.1)	3 (15.0)
*95% CI* ^*a*^	0.6, 16.7	7.6, 34.5	15.8, 75.0	2.5, 27.8	5.2, 36.0
**Adverse events leading to treatment discontinuation**					
*n (%)*	0	2 (6.9)	0	2 (9.1)	1 (5.0)
*95% CI* ^*a*^	0.0, 11.4	1.9, 22.0	0.0, 35.4	2.5, 27.8	0.9, 23.6

CI = confidence interval; CLCR = creatinine clearance; MedDRA = medical dictionary for regulatory activities; MiRI = mild renal impairment; SRI = severe renal impairment.

^a^Score method.

Two deaths were reported in patients with SRI receiving edoxaban 15 mg. One patient experienced acute cardiac failure on the day after the initiation of study treatment, which resulted in death the same day. The second death was due to cardiac failure experienced 27 days after the completion of study treatment; the patient died 2 days later. Both deaths were considered unrelated to the study drug. A total of 8 serious AEs other than death were reported: 1 occurred in a patient with MiRI receiving edoxaban 30 mg, 4 occurred in 3 patients with SRI receiving edoxaban 15 mg, and 3 occurred in 3 patients with SRI receiving fondaparinux 1.5 mg. Serious AEs included dementia, cerebral infarction, pyelonephritis, and femur fracture in the group with SRI receiving edoxaban 15 mg. A joint dislocation occurred in a patient with MiRI receiving edoxaban 30 mg. Sepsis, cerebral infarction, and a periprosthetic fracture occurred in 1 patient each with SRI receiving fondaparinux 1.5 mg. None of the serious AEs were considered related to study drug.

Three patients discontinued treatment due to an AE. In the SRI edoxaban 15-mg group (CL_CR_ ≥20 to <30 mL/min), these included the above-mentioned cardiac failure, along with a subcutaneous hematoma reported in a second patient. The third AE-related discontinuation was a cerebral infarction, which occurred in a patient with SRI in the fondaparinux group.

### Plasma concentrations

**Figure 3:**
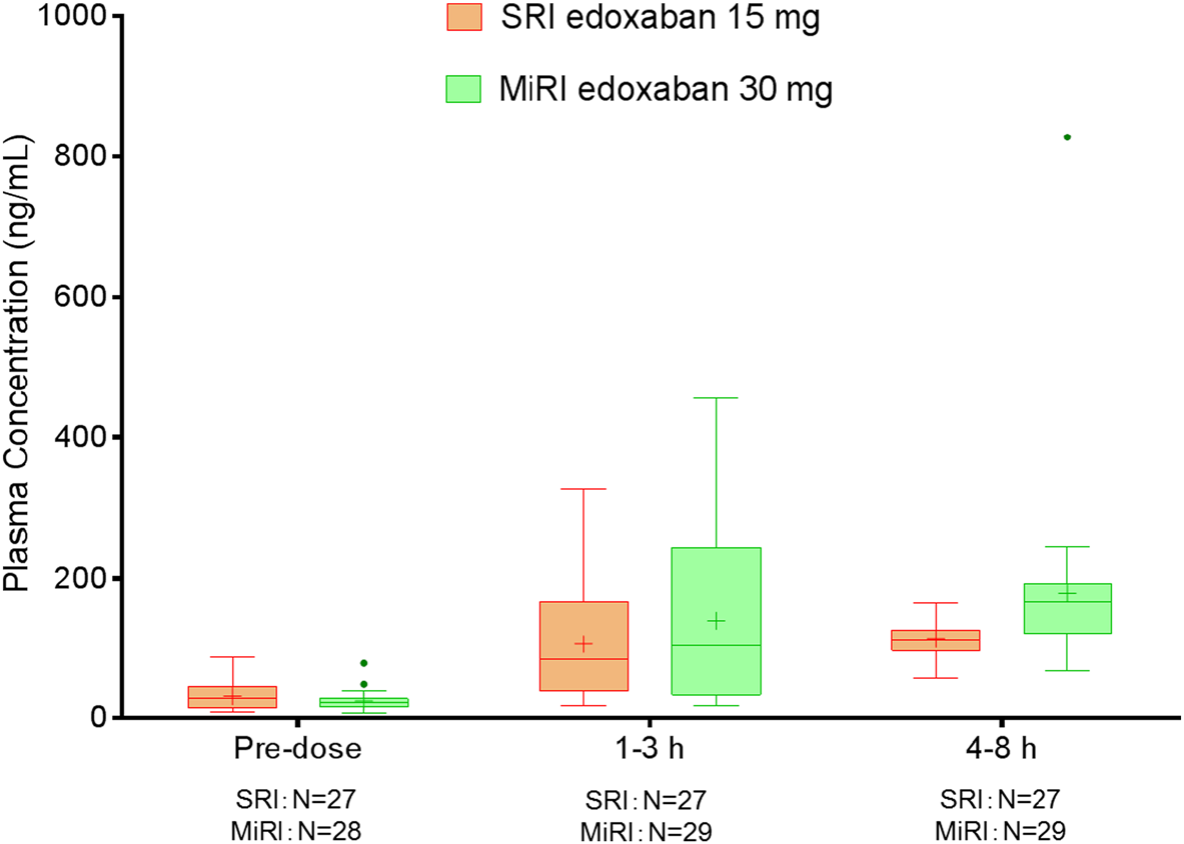
**Mean plasma concentrations of edoxaban on day 7.** Horizontal line = median; + = mean; error bars = minimum and maximum values within 1.5 interquartile range; • = outliers; SRI = severe renal impairment; MiRI = mild renal impairment.

On day 7, plasma concentrations of edoxaban in patients with SRI receiving a 15-mg dose overlapped considerably with MiRI patients receiving a 30-mg dose (Figure 3). Compared with patients with MiRI who received edoxaban 30 mg, the mean (SD) edoxaban plasma concentration in patients with SRI who received edoxaban 15 mg was 27% higher at predose (31.4 [18.6] vs 24.7 [14.1] ng/mL, respectively); 24% lower at 1 to 3 hours postdose (106 [75.8] vs 140 [120] ng/mL, respectively); and 37% lower at 4 to 8 hours postdose (113 [24.9] vs 179 [132] ng/mL). Within the SRI edoxaban 15-mg group, those with CL_CR_ ≥15 to <20 mL/min had a 2-fold higher mean edoxaban plasma concentration compared to those with CL_CR_ ≥20 to <30 mL/min at predose on day 7 (50.7 [18.1] vs 24.6 [13.6]); however, mean concentrations were comparable at 1 to 3 hours postdose (106 [72.9] vs 106 [78.7], respectively) and 4 to 8 hours postdose (126 [18.7] vs 109 [25.8], respectively). Edoxaban plasma concentrations on the completion day of study treatment were similar to those on day 7.

### Biomarkers

#### PT

**Figure 4:**
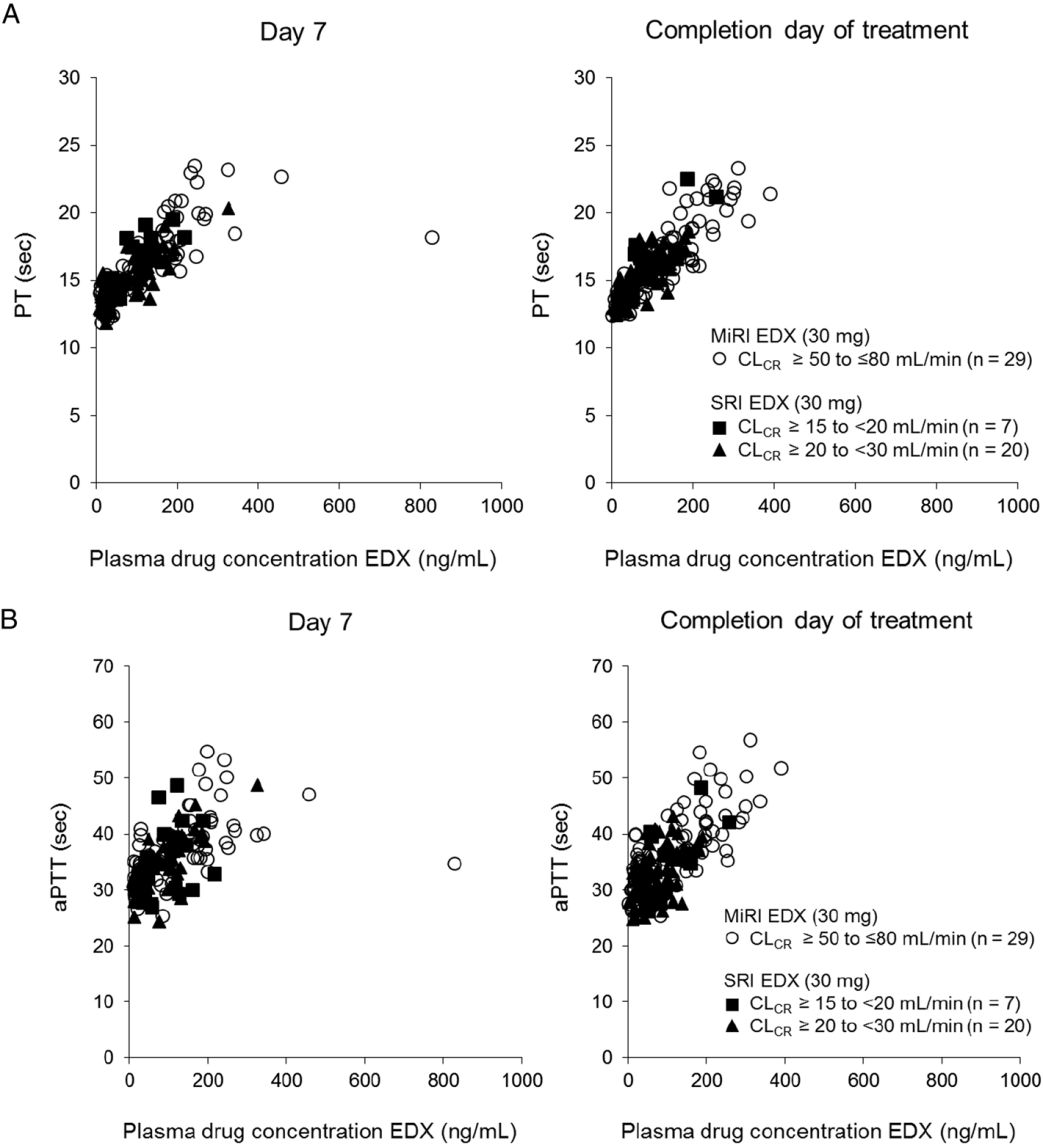
**Relationship between plasma edoxaban concentrations and A) PT; B) aPTT on day 7 and completion.** aPTT = activated partial thromboplastin time; CL_CR_ = creatinine clearance; EDX = edoxaban; MiRI = mild renal impairment; PT = prothrombin time; SRI = severe renal impairment.

On day 7, mean PT in SRI patients receiving edoxaban 15 mg was comparable to that of MiRI patients receiving edoxaban 30 mg at predose (14.0 vs 13.7 seconds, respectively), 1 to 3 hours postdose (16.1 vs 16.8 seconds, respectively) and 4 to 8 hours postdose (16.2 vs 17.4 seconds, respectively). For SRI patients receiving fondaparinux 1.5 mg, mean PT was 13.5 seconds at predose and there was no marked prolongation of mean PT at 1 to 3 hours (13.6 seconds) or 4 to 8 hours (13.5 seconds) postdose. A similar time course of mean PT was observed on the completion day of study treatment to that of day 7 in all treatment groups (Table 4). The relationship between PT and plasma edoxaban concentration in patients with SRI receiving edoxaban 15 mg was similar to that in patients with MiRI receiving edoxaban 30 mg (Figure 4A). Mean PT was prolonged as plasma edoxaban concentration increased on both day 7 and at the completion of study treatment.

**Table 4:** **Mean PT and aPTT values by treatment group on day 7 and at treatment completion**

			**Day 7**			**Completion day of treatment**		
**Parameter**		**Treatment group**	**Pre dose**	**1 to 3 h**	**4 to 8 h**	**Pre dose**	**1 to 3 h**	**4 to 8 h**
PT (s)	**MiRI** *n*	Edoxaban 30 mg	29	29	29	30	29	28
	*Mean (SD)*	CL_CR_ ≥50 to <80 mL/min	13.7 (1.1)	16.8 (3.6)	17.4 (1.8)	13.6 (1.0)	17.6 (3.4)	17.5 (2.1)
	**SRI** *n*	Edoxaban 15 mg (total)	27	27	27	26	25	26
	*Mean (SD)*	CL_CR_ ≥15 to <30 mL/min	14.0 (1.2)	16.1 (2.1)	16.2 (1.4)	14.1 (1.1)	15.9 (1.9)	16.5 (1.6)
		Edoxaban 15 mg	7	7	7	6	7	7
		CL_CR_ ≥15 to <20 mL/min	14.8 (1.3)	16.9 (2.1)	16.9 (1.1)	15.0 (1.5)	16.6 (2.3)	17.1 (2.5)
		Edoxaban 15 mg	20	20	20	20	18	19
		CL_CR_ ≥20 to <30 mL/min	13.8 (1.0)	15.8 (2.1)	15.9 (1.4)	13.9 (0.9)	15.6 (1.8)	16.3 (1.2)
		Fondaparinux	20	20	20	19	17	18
		CL_CR_ ≥20 to <30 mL/min	13.5 (0.9)	13.6 (0.9)	13.5 (0.9)	13.7 (1.1)	13.7 (1.1)	13.6 (1.1)
aPTT (s)	**MiRI** *n*	Edoxaban 30 mg	29	29	29	30	29	28
	*Mean (SD)*	CL_CR_ ≥50 to <80 mL/min	32.2 (3.3)	37.7 (6.2)	38.9 (6.3)	31.7 (3.6)	39.3 (7.5)	39.8 (6.5)
	**SRI** *n*	Edoxaban 15 mg (total)	27	27	27	26	25	26
	*Mean (SD)*	CL_CR_ ≥15 to <30 mL/min	31.6 (3.5)	35.7 (6.0)	36.1 (4.2)	31.0 (4.3)	34.0 (4.8)	35.6 (4.8)
		Edoxaban 15 mg	7	7	7	6	7	7
		CL_CR_ ≥15 to <20 mL/min	32.7 (4.4)	36.6 (7.3)	36.2 (7.0)	31.1 (4.9)	34.2 (6.0)	34.7 (7.0)
		Edoxaban 15 mg	20	20	20	20	18	19
		CL_CR_ ≥20 to <30 mL/min	31.2 (3.1)	35.4 (5.7)	36.1 (3.0)	31.0 (4.2)	34.0 (4.4)	36.0 (3.8)
		Fondaparinux	20	20	20	19	17	18
		CL_CR_ ≥20 to <30 mL/min	32.5 (4.4)	32.6 (4.5)	32.3 (5.0)	32.9 (3.9)	33.2 (5.8)	33.5 (4.7)

aPTT = activated partial thromboplastin time; CL_CR_ = creatinine clearance; MiRI = mild renal impairment; PT = prothrombin time; SD = standard deviation; SRI = severe renal impairment.

#### aPTT

The time course of mean aPTT observed on day 7 and on the completion day of treatment was also similar to those seen for mean PT in all treatment groups (Table 4). Mean aPTT was prolonged at 1 to 3 hours and 4 to 8 hours postdose on day 7 in all edoxaban treatment groups, while no prolongation was seen in the fondaparinux treatment group. A similar time course of mean aPTT was observed on the completion day of study treatment compared with that of day 7 in all treatment groups (Table 4). The relationship between aPTT and plasma edoxaban concentration in patients with SRI receiving edoxaban 15 mg was also similar to that in patients with MiRI receiving edoxaban 30 mg. On both day 7 and the completion day of treatment, aPTT was prolonged as plasma edoxaban concentration increased (Figure 4B).

### Thromboembolic events

No thromboembolic events occurred in any treatment group during the duration of the study.

## Discussion

This study evaluated the safety and relationship between plasma concentration and coagulation biomarkers following edoxaban 15 mg once daily dosing in patients with SRI undergoing TKA, THA, or HFS. The incidence of bleeding events were similar between patients with SRI who received edoxaban 15 mg once daily compared with patients with MiRI who received edoxaban 30 mg once daily. Likewise, among patients with SRI who received either edoxaban 15 mg or fondaparinux 1.5 mg once daily, major or CRNM bleeding rates were similar. Of interest, no major bleeding events occurred during this study in any treatment group. Minor bleeding rates appeared to be lower in the SRI edoxaban 15-mg group compared with both the MiRI edoxaban 30-mg and SRI fondaparinux groups. Edoxaban plasma concentrations did differ between the groups from 24% to 37% at different timepoints, but no notable differences on mean PT and aPTT levels were observed.

Overall, bleeding rates observed in the current study are similar to those reported in phase 3 studies that evaluated the safety and efficacy of edoxaban 30 mg once daily for the prevention of VTE in Japanese patients undergoing THA, TKA, or HFS [20-22]. For example, in the current study, the incidence of major or CRNM bleeding was 6.7% in the MiRI edoxaban 30-mg group and 3.4% in the SRI edoxaban 15-mg group. In the phase 3 THA [20], TKA [22], and HFS [21] studies, these rates were 2.6%, 6.2%, and 3.4%, respectively. Patients with SRI were excluded from these studies.

The clinical results of the current study are supported by edoxaban plasma concentration and coagulation biomarker results. While mean edoxaban plasma concentrations differed between patients in the SRI 15-mg group and the MiRI 30-mg group at each time point on day 7, the range of individual measures overlapped considerably, suggesting similar exposure. In addition, edoxaban-induced prolongation of PT and aPTT were similar between the two edoxaban treatment groups. The observed prolongation of PT and aPTT correlated with individual edoxaban plasma concentrations; as the plasma concentrations increased, PT and aPTT were prolonged and this concentration/biomarker relationship was not affected by the severity of renal impairment. These relationships were consistent with previously documented data from healthy volunteers [13] and patients undergoing THA [20] or HFS [21].

There were no reports of thromboembolic events during this study. All patients enrolled utilized physiotherapy, which may have increased the thromboprophylactic potential of treatment. The combination of pharmacologic and mechanical thromboembolic prophylaxis has been shown to further reduce the occurrence of thromboembolic events versus compression or pharmacologic prophylaxis alone [23], and this combination therapy is recommended by the Japanese Circulation Society for patients at the highest risk for developing thromboembolisms [4].

Serious AEs occurred in 16% of patients with SRI, compared with 3% of those with MiRI. This is not unexpected, as patients with decreasing renal function have increasing comorbidity burdens [24,25]. Furthermore, none of the serious AEs reported in this study were considered related to study drug.

A potential limitation of this study is the small number of patients enrolled, which does not provide enough power to determine any statistically significant differences in the rate of bleeding events between treatment groups or to evaluate efficacy. However, the objective of this study was to evaluate the safety of the edoxaban 15 mg once daily dose in patients with SRI undergoing LLOS, not to compare treatments. The data presented here suggest that edoxaban 15 mg once daily is well tolerated in this patient population, but larger studies would be needed to confirm these results and provide data regarding efficacy.

## Conclusions

The pharmacologic options available in Japan for the prevention of VTE in patients with SRI undergoing LLOS are limited. As previously mentioned, earlier phase 3 studies leading to the approval of edoxaban 30 mg for the prevention of thromboembolic events excluded patients with SRI and thus edoxaban is not presently approved for use in that patient population. The results presented here suggest that edoxaban 15 mg does not increase the risk for bleeding in Japanese patients with SRI undergoing LLOS and may be an option for thromboprophylaxis, however this requires confirmation in a larger study.

## Appendix

Safety Assessment Committee: Fujita S, Kawai Y. Efficacy Assessment Committee: Fujita S. Study Investigators: Tsuji M, Kikuchi T, Hashimoto F, Seki H, Owaki H, Nakai T, Hayashi M, Shiota N, Teramoto H, Nishida K, Miyahara H, Doiguchi Y, Nakano T, Fukumoto Y, Mori H, Arakaki A. Sponsor Representatives: Kimura T, Fukuzawa M (Project Leader/Study Leader), Abe K (statistician).

## Abbreviations

AEs: Adverse events
ALT: Alanine aminotransferase
AST: Aspartate aminotransferase
aPTT: Activated partial thromboplastin time
CI: Confidence interval
CL_CR_: Creatinine clearance
CRNM: Clinically relevant non-major
CT: Computed tomography
DVT: Deep venous thromboembolism
EDX: Edoxaban
FPX: Fondaparinux
GCP: Good Clinical Practice
HFS: Hip fracture surgery
LLOS: Lower-limb orthopedic surgery
MedDRA: Medical dictionary regulatory activities
MiRI: Mild renal impairment
NSAID: Nonsteroidal anti-inflammatory drugs
PE: Pulmonary embolism
P-gp: P-glycoprotein
PK: Pharmacokinetics
PT: Prothrombin time
SD: Standard deviation
SRI: Severe renal impairment
THA: Total hip arthroplasty
TKA: Total knee arthroplasty
ULN: Upper limit of normal
VTE: Venous thromboembolism
